# Enhanced Mitochondrial Dynamics and Reactive Oxygen Species Levels with Reduced Antioxidant Defenses in Human Epicardial Adipose Tissue

**DOI:** 10.3390/metabo15070481

**Published:** 2025-07-16

**Authors:** Ana Burgeiro, Diana Santos, Ana Catarina R. G. Fonseca, Inês Baldeiras, Ermelindo C. Leal, João Moura, João Costa-Nunes, Patrícia Monteiro Seraphim, Aryane Oliveira, António Canotilho, Gonçalo Coutinho, David Prieto, Pedro Antunes, Manuel Antunes, Eugenia Carvalho

**Affiliations:** 1 CNC—Center for Neuroscience and Cell Biology, University of Coimbra, 3004-504 Coimbra, Portugalecleal@cnc.uc.pt (E.C.L.); jmouraalves@gmail.com (J.M.); jpcosta.nunes@gmail.com (J.C.-N.); aryanecruz.op@gmail.com (A.O.); 2 CiBB—Center for Innovative Biomedicine and Biotechnology, University of Coimbra, 3004-504 Coimbra, Portugal; 3 Local Health Unit of the Leiria Region, E.P.E, 2140-197 Leiria, Portugal; 4 PhD Programme in Experimental Biology and Biomedicine (PDBEB), Institute for Interdisciplinary Research, University of Coimbra, 3030-789 Coimbra, Portugal; 5 Institute for Interdisciplinary Research, University of Coimbra, 3030-789 Coimbra, Portugal; 6 Department of Physiotherapy, School of Sciences and Technology, Campus Presidente Prudente, Sao Paulo State University (UNESP), Sao Paulo 19060-900, Brazil; 7 Cardiothoracic Surgery Unit, University Hospital of Coimbra, 3000-075 Coimbra, Portugal; 8 University Clinic for Cardiothoracic Surgery, Faculty of Medicine, University Hospital of Coimbra, 3000-548 Coimbra, Portugal

**Keywords:** epicardial adipose tissue, subcutaneous adipose tissue, mitochondrial dynamics, oxidative stress, antioxidant defense, diabetes mellitus, coronary artery disease

## Abstract

**Background/Objectives**: Epicardial adipose tissue (EAT) is metabolically active and is in dynamic crosstalk with the surrounding cardiomyocytes, modulating their function and metabolism. Oxidative stress is a key contributor to cell death and cardiac remodeling, is a hallmark of diabetes (DM) and cardiovascular disease, such as coronary artery disease (CAD). However, little is known about these processes in EAT from patients undergoing cardiac surgery. This study investigates changes in mitochondrial dynamics, reactive oxygen species (ROS) production, and antioxidant defense levels in EAT compared to subcutaneous adipose tissue (SAT) in patients undergoing cardiac surgery, with a focus on the impact of DM and CAD. **Methods**: Adipose tissue biopsies were collected from 128 patients undergoing surgical cardiac intervention. Mitochondrial dynamics and oxidative stress markers were analyzed. **Results**: EAT exhibited increased expression of mitochondrial fusion markers [mitofusin 1 (*p* ≤ 0.001), mitofusin 2 (*p* = 0.038), and optic atrophy 1 (*p* ≤ 0.001)], as well as fission markers [fission 1 *(p* ≤ 0.001) and dynamin-related protein 1 (*p* ≤ 0.001)] relative to SAT. Additionally, ROS levels (dihydroethidium, *p* = 0.004) were elevated, while lipid peroxidation (malondialdehyde, *p* ≤ 0.001) was reduced in EAT compared to SAT. Reduced glutathione (GSH) levels (*p* ≤ 0.001) and the redox buffer ratio between reduced and oxidized glutathione (GSH/GSSG, *p* ≤ 0.001) were significantly increased in EAT. Interestingly, glutathione peroxidase activity (*p* ≤ 0.001) and the antioxidant defense markers catalase (*p* ≤ 0.001) and superoxide dismutase 2 (*p* = 0.001) were significantly reduced in EAT compared to SAT. **Conclusions**: The findings provide a unique molecular insight into the mitochondrial dynamics and oxidative stress profiles of EAT, highlighting potential avenues for a novel diagnostic method and therapeutic strategies for cardiac disease.

## 1. Introduction

Cardiovascular diseases (CVDs) are the most incapacitating and fatal diseases worldwide. Among them, coronary artery disease (CAD) is the most prevalent and detrimental condition [1]. In addition, almost two-thirds of patients with CVD have abnormal glucose homeostasis [2] and the prevalence of CAD in subjects with DM is about 12% [3].

Epicardial adipose tissue (EAT) is a specialized and active fat depot located around the coronary arteries, the visceral pericardium, and myocardium [4]. Compared with other fat deposits, it presents rapid metabolism, with high thermogenic capacity, and a unique transcriptome and secretome [5,6,7,8,9]. Moreover, EAT can impact the local cardiac structure and function due to its characteristic energetics and close cellular crosstalk with neighboring endothelial cells and cardiomyocytes [10].

Mitochondria biogenesis, maintenance, and turnover are modulated by finely tuned regulatory networks [11]. Moreover, mitochondria malfunction has been implicated in the development of aging [12], inherited genetic diseases [13], type 2 diabetes mellitus (DM) [14] and CVD [15,16]. However, while altered mitochondrial dynamics have been extensively described in cardiomyocytes of the failing heart [16,17,18], they are poorly studied in EAT. Thus, in the present study, we evaluated the following: (1) whether the mitochondrial fusion/fission mechanisms along with oxidative stress markers, that include reactive oxygen species (ROS) and lipid peroxidation, as well as antioxidant defenses, mediated by glutathione (GSH), catalase, and superoxide dismutase (SOD) were altered in EAT compared to SAT; (2) the potential impact of DM and CAD on the described mitochondrial mechanisms comparing the two adipose tissues, and finally (3) how DM and CAD may influence mitochondrial dynamics in EAT, in patients undergoing cardiac surgery.

## 2. Materials and Methods

### 2.1. Adipose Tissue Donors

Study participants (*n* = 128; 99 males and 29 females) with a well-defined set of preoperative variables such as age, hypertension, dyslipidemia, smoking habits, body mass index, and family history of heart disease, were recruited in collaboration with the Cardiothoracic Surgery Unit at the University Hospital of Coimbra, in accordance with our previous studies [8,9,19]. Paired SAT and EAT biopsies were obtained from the same patients during elective coronary artery bypass grafting (CABG), valve repair or valve replacement, or patients undergoing both surgeries. Patients with CAD subjected, at least, to a CABG were considered as the CAD group, while patients subjected to only a valvular surgery were considered as the NCAD group. Tissue samples were consistently collected after pericardial opening and heart exposure, but before the initiation of a cardiopulmonary bypass or surgical manipulation and were obtained from the proximal right coronary artery (EAT) and the sternum region (SAT), as described previously [8,9]. The study was performed after receiving consent from participants and approved by the Ethical Committee of the Coimbra University Hospital Centre (HUC-35-11 and OBS.SF.24-2021). The studies were carried out according to the Declaration of Helsinki.

### 2.2. Adipose Tissue Gene Expression

Total RNA extracted from EAT and SAT biopsies was isolated using the RNeasy MiniKit (Germantown, MD, USA), and the concentration was determined by OD 260 measured using a NanoDrop 1000 spectrophotometer (ThermoScientific, Waltham, MA, USA). cDNA was synthesized from 1 μg of RNA using the Applied Biosystems High-Capacity cDNA Reverse Transcriptase kit (Forest City, CA, USA). Briefly, 2 μL of 10× RT Buffer, 0.8 μL of 25x dNTP Mix, 2 μL of 10x RT random primers, 1 μL of MultiScribe Reverse Transcriptase, and 4.2 μL of nuclease free H2O were added to 10 μL of RNA (1 μg) sample. Quantitative real-time PCR reaction was then performed in a Bio-Rad iCycler iQ5 (Hercules, CA, USA). For each reaction, 10 μL volume were used containing 2.5 μL cDNA, 5 μL 2x with SYBRGreen Supermix (Quanta Biosciences, Gaithersburg, MA, USA), 1 μL of each primer (250 nM), and 0.5 μL of H_2_O PCR grade. PCR primers were designed using Beacon Designer software and synthesized by IDT-Integrated DNA Technologies, Inc. (BVBA, Leuven, Belgium), and described in Table 1. Relative quantification was determined by the 2^−ΔCt^ method and normalized to the β-actin, according to our previous studies [8,9].

### 2.3. Adipose Tissue Protein Expression

EAT and SAT biopsies were homogenized in radioimmunoprecipitation assay (RIPA) buffer (50 mMTris–HCl buffer pH 7.5, 150 mM NaCl, 1% Triton X-100, 0.5% sodium deoxycholate, 0.1% sodium dodecyl sulfate, 5 mM ethylene glycol tetraacetic acid, protease inhibitor cocktail, phosphatase inhibitor cocktail, and 1 mM dithiothreitol). Protein concentrations were determined by the bicinchoninic acid (BCA) method (Pierce^®^ BCA Protein Assay Kit, Thermo Scientific, Rockford, IL, USA). Total protein extracts were denatured at 95 °C for 5 min in 6× sample buffer [0.35 M Tris–HCl, pH 6.8, 30% glycerol, 10% sodium dodecyl sulfate, 0.6 M dithiothreitol, 0.03% bromophenol blue]. Equal protein amounts (30 μg) were separated on 7.5% or 10% SDS-PAGE gels, followed by transfer to PVDF membranes (Millipore Chemicon, MA, USA). After blocking, membranes were incubated overnight at 4 °C with primary antibodies against peroxisome proliferator-activated receptor gamma co-activator-1 alpha (PGC1-α, 1:500 dilution, Santa Cruz Biotechnology Dallas, TX, USA) β-actin (I-19, 1:3000 dilution, Santa Cruz Biotechnology, Dallas, TX, USA), catalase (1:5000 dilution, Merck KGaA, Darmstadt, Germany) and SOD2 (1:1000 dilution, Millipore Chemicon, Temecula, CA, USA). After incubation, the membranes were washed and incubated for 1 h at room temperature with anti-rabbit (1:10,000) secondary antibodies (ThermoFisher Scientific, Waltham, MA, USA) against all the primary antibodies, except for actin, were conjugated to horseradish peroxidase, and immune complexes were detected using enhanced Chemiluminescence reagent (ThermoFisher Scientific, Waltham, MA, USA). The anti-rabbit (1:5000) secondary antibodies Secondary antibody (Santa Cruz Biotechnology, Dallas, TX, USA) for actin was conjugated to alkaline phosphatase and immune complexes were detected using Enhanced Chemifluorescence reagent (Amersham Pharmacia Biotech, Buckinghamshire, UK). Bands were visualized using the enhanced chemifluorescence (ECF) substrate kit (Amersham, GE HealthCare, Buckinghamshire, UK) on the ChemidocTM Touch system (Bio-Rad Laboratories, Hercules, CA, USA) and quantified using Image Lab version 5.2.1 build 11 (Bio-Rad Laboratories, Hercules, CA, USA). β-Actin was used as a loading control.

### 2.4. Oxidative Stress Quantification

Oxidative stress levels and antioxidant capacity in EAT and SAT biopsies were assessed either in tissue cross-sections or following homogenization in RIPA buffer (50 mM Tris–HCl, pH 7.5; 150 mM NaCl; 1% Triton X-100; 0.5% sodium deoxycholate; 0.1% SDS; 5 mM EGTA; 1 mM DTT).

#### 2.4.1. Reactive Oxygen Species Evaluation

The dihydroethidium (DHE) assay was performed to detect and measure the production of ROS in EAT and SAT biopsies previously immersed in the Optimal Cutting Temperature compound (OCT, VWR, Porto, Portugal) and immediately frozen at −80 °C. Cross-sections of 30 µm thickness were thawed and incubated with 1 µM DHE (Sigma-Aldrich, Saint Louis, MO, USA) dissolved in PBS (pH 7.4), at 37 °C for 30 min, in the dark. Afterwards, slides were washed twice with PBS, fixed with 4% paraformaldehyde in PBS for 5 min and stained with DAPI (1:1000, Sigma-Aldrich, Saint Louis, MO, USA) for 5 min. Slides were mounted (Entellan^®^, Merck KGaA, Darmstadt, Germany) and covered with a coverslip and immediately observed using a confocal microscope (Carl Zeiss LSM 710, Jena, Germany). The DHE fluorescence corresponds to the ability of reactive oxygen species, particularly superoxide, to oxidize DHE, leading to the production of ethidium bromide, that intercalates with DNA in the nucleus, emitting nuclear red fluorescence. The DHE fluorescence was quantified using Fiji software (Fiji is just ImageJ2 version 2.9.0/1.53t). Image J software (National Institute Health, Bethesda, MD, USA) and it was normalized to the number of cells in the corresponding image. Then, the average was calculated using normalized DHE fluorescence obtained on the 10 highest resolution fields (200x).

#### 2.4.2. Lipid Peroxidation

Lipid peroxidation in EAT and SAT homogenates was assessed by fluorometric determination (excitation at 515 nm and emission at 553 nm; FP-2020/2025, Jasco, Tokyo, Japan) of malondialdehyde (MDA) combined with high-performance liquid chromatography (HPLC, Gilson, Lewis Center, OH, USA), using the ClinRep complete kit (RECIPE, Munich, 228 Germany). Evaluation of the chromatograms was performed via peak areas that are proportional to the MDA concentration, obtained from EAT and SAT homogenates and expressed in micromoles of MDA per gram of protein (μmol/g protein).

#### 2.4.3. Reduction and Oxidation of Glutathione

Reduced (GSH) and oxidized glutathione (GSSG) levels in EAT and SAT homogenates were evaluated by HPLC (Gilson, Lewis Center, OH, USA) with fluorimetric detection (excitation at 385 nm and emission at 515 nm; FP-2020/2025, Jasco, Tokyo, Japan), using the Immunodiagnostik kit (Immunodiagnostik AG, Bensheim, Germany), as previously described [20]. GSH and GSSG were separated according to retention time and quantified by chromatographic peak area. Results were expressed as micromoles of either GSH or GSSG per gram of protein (μmol/g protein).

#### 2.4.4. Glutathione Peroxidase Activation

The activity of glutathione peroxidase (GPx) in EAT and SAT homogenates was evaluated by spectrophotometry using tert-butylperoxide (Sigma-Aldrich, Saint Louis, MO, USA) as a substrate [21], monitoring oxidized glutathione levels through the quantification of NADPH (Sigma) oxidation at 340 nm and at 37 °C, in a thermostated spectrophotometer (UVIKON 933 UV/Visible, Kontron Instruments, Milan, Italy). Results were expressed in international units of enzyme per gram of protein (U/g).

#### 2.4.5. Glutathione Reductase Activation

Glutathione reductase (GRed) activity in EAT and SAT homogenates was evaluated by spectrophotometry at 340 nm, [22] using GSSG (Sigma -Aldrich, Saint Louis, MO, USA) as a substrate and monitoring its reduction to GSH through the quantification of NADPH (Sigma-Aldrich, Saint Louis, MO, USA) oxidation at 37 °C in a thermostated spectrophotometer (UVIKON 933 UV/Visible, Kontron Instruments, Milan, Italy). GRed activity was expressed in international units of enzyme per gram of protein (U/g).

### 2.5. Statistical Analysis

Normality was assessed using the Shapiro–Wilk test, and variance homogeneity with Levene’s test. For non-normally distributed data, the Wilcoxon signed-rank test was applied for paired comparisons, and the Mann–Whitney U test for unpaired data. When technical limitations prevented pairing, the Mann–Whitney test was used instead. Categorical variables were analyzed using the χ^2^ test. Results are presented as mean ± standard error of the mean (SEM) or median (interquartile range, Q1–Q3), as appropriate. All statistical analyses were performed in SPSS (version 28), with a significance threshold of *p* < 0.05. Graphs were generated using GraphPad Prism (version 8, GraphPad Inc., La Jolla, CA, USA).

## 3. Results

### 3.1. Characteristics of the Study Population

A group of 128 male (n = 99) and female (n = 29) patients were recruited and stratified according to the presence or absence of DM. Thus, 68 patients without DM (NDM group) and 60 patients with DM (DM group) were included. Hypertension (*p* = 0.012) was present in 82% of patients with DM, that also presented higher BMI (*p* = 0.030), as shown in Table 2. Moreover, besides DM-related medication (insulin and/or oral antidiabetics), the statin intake (*p* = 0.008) was the only medication that differed between the two groups, as seen in Table 2. No differences were observed regarding sex, age, or the incidence of cardiovascular risk factors, when comparing both groups. Importantly, patients were also stratified according to cardiac pathology, either NCAD (n = 67) or CAD (n = 61). A significantly number of male patients (*p* = 0.001) had CAD (*n* = 55, 90%) compared to the NCAD group (n = 44, 66%). Moreover, key CVD contributing risk factors, such as hypertension (*p* = 0.015, 74% versus 63%), dyslipidemia (*p* ≤ 0.001, 87% versus 60%) and family history of heart disease (*p* ≤ 0.001, 74% versus 63%) were more prevalent int the patients with CAD compared to the NCAD groups. Furthermore, the parentage of patients with CAD group with smoking habits is significantly increased compared to the NCAD group, (*p* = 0.025, 44% versus 25%). However no further differences were observed when comparing according to the different subgroups, as presented in Table 2. The administration of antiplatelet medication (*p* ≤ 0.001, 50% versus 22%), β blocker (*p* = 0.015, 70% versus 47%), calcium channel blockers (*p* = 0.014, 23% versus 8%), statins, (*p* ≤ 0.001, 85% versus 57%) and vasodilators (*p* = 0.004, 31% versus 10%) was increased in the CAD group compared to the NCAD group, as seen in Table 2.

Demographics and clinical characteristics according to cardiac pathology (NCAD and CAD) were described according to the presence or absence of DM, described in Table S1. Briefly, from the 67 patients in the NCAD, 31 patients (47%) had DM, and in the CAD group, 29 patients had DM.

### 3.2. Mitochondrial Fusion and Fission Are Increased in Epicardial Adipose Tissue

**Figure 1:**
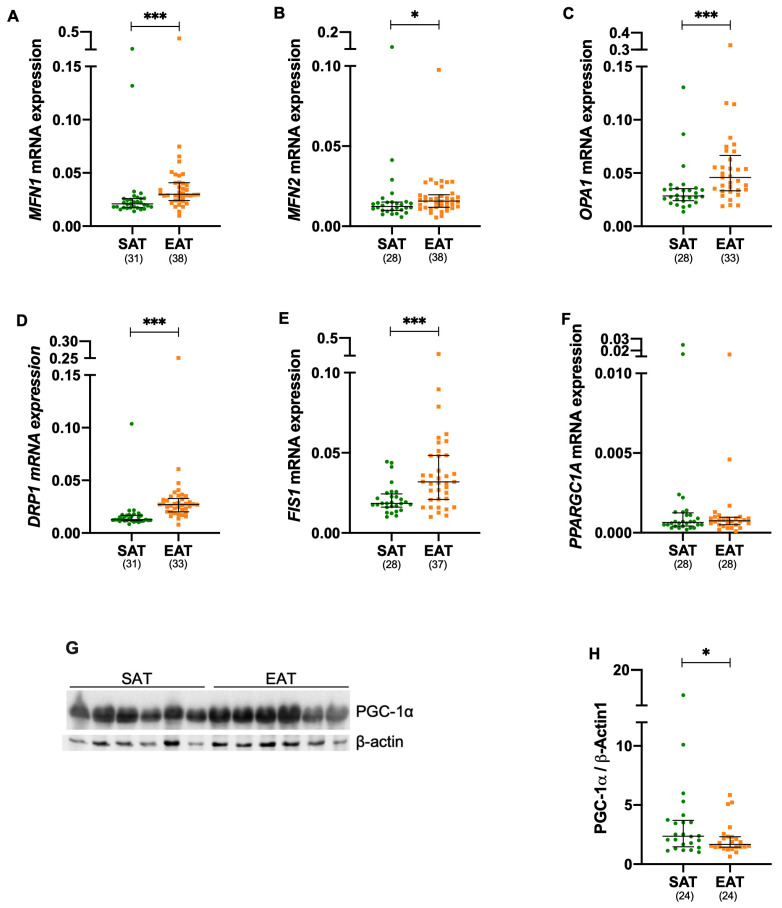
Mitochondrial fusion and fission mechanisms are significantly enhanced in epicardial adipose. The mRNA expression levels of *MFN1* (**A**), *MFN2* (**B**), *OPA1* (**C**), *DRP1* (**D**), *FIS1* (**E**), and *PPARGC1A* (**F**), along with PGC-1α protein levels (**G**,**H**) were analyzed. Gene expression was quantified by RT-qPCR, and protein expression by Western blot, with normalization to the housekeeping gene or protein β-actin, respectively. The number of patients included in each assay is indicated. A *p* ≤ 0.05 was considered statistically different, * *p* ≤ 0.05; *** *p* ≤ 0.001. EAT, epicardial adipose tissue; DRP1, dynamin-related protein 1; FIS1, fission 1 MFN1, mitofusin 1; MFN2, mitofusin 2; OPA1, optic atrophy 1; PGC-1α, peroxisome proliferator-activated receptor gamma co-activator-1 alpha for gene and protein, respectively; SAT subcutaneous adipose tissue.

Gene and protein expression in EAT was compared to SAT from all patients. Thus, gene expression of mitochondrial fusion proteins [mitofusin 1 (*MFN1*, *p* ≤ 0.001), mitofusin 2 (*MFN2*, *p* = 0.038)], and optic atrophy 1 (*OPA1*, *p* ≤ 0.001)] was significantly increased in EAT, as seen in Figure 1A–C, respectively, and Table S2. Similarly, gene expression of the mitochondrial fission proteins, dynamin-related protein 1 (*DRP1*, *p* ≤ 0.001), and fission protein 1 (*FIS1*, *p* ≤ 0.001), was also elevated in the EAT, as seen in Figure 1D,E, respectively, and Table S2. Interestingly, while there were no differences in peroxisome proliferator-activated receptor gamma co-activator-1 alpha (*PPARGC1A*) gene expression, as seen in Figure 1F, and Table S2, a significant reduction in PCG1-α protein levels (*p* = 0.015) was observed in EAT, as shown in Figure 1G,H and Table S2.

Detailed expression profiles between all proteins after stratification for DM and CAD are described in Table S3. Briefly, gene expression was consistently increased in EAT compared to the respective SAT for *MFN1* (NDM: *p* = 0.011; DM: *p* ≤ 0.001; NCAD: *p* ≤ 0.001, and CAD: *p* = 0.017) and *DRP1* (NDM: *p* ≤ 0.011; DM: *p* ≤ 0.001; NCAD: *p* ≤ 0.001, and CAD: *p* ≤ 0.017). Furthermore, EAT showed an increased expression of OPA1 EAT in the NDM (*p* = 0.017), DM (*p* = 0.013), and NCAD group (*p* = 0.002), while *FIS1* was upregulated in the NDM (*p* = 0.012), DM (*p* = 0.040), and NCAD group (*p* ≤ 0.001). Moreover, *MFN2* expression was significantly increased in EAT, but only within the NCAD group (*p* ≤ 0.011), while PCG1-α protein levels were significantly reduced exclusively in EAT of the DM group (*p* = 0.016), Table S3.

No further differences were observed in mitochondrial markers within EAT based on the presence of DM (EAT NDM versus EAT DM) or CAD (EAT NCAD versus EAT CAD), as presented in Table S3.

### 3.3. Oxidative Stress Is Increased in Epicardial Adipose Tissue Under Cardiac Disease

**Figure 2:**
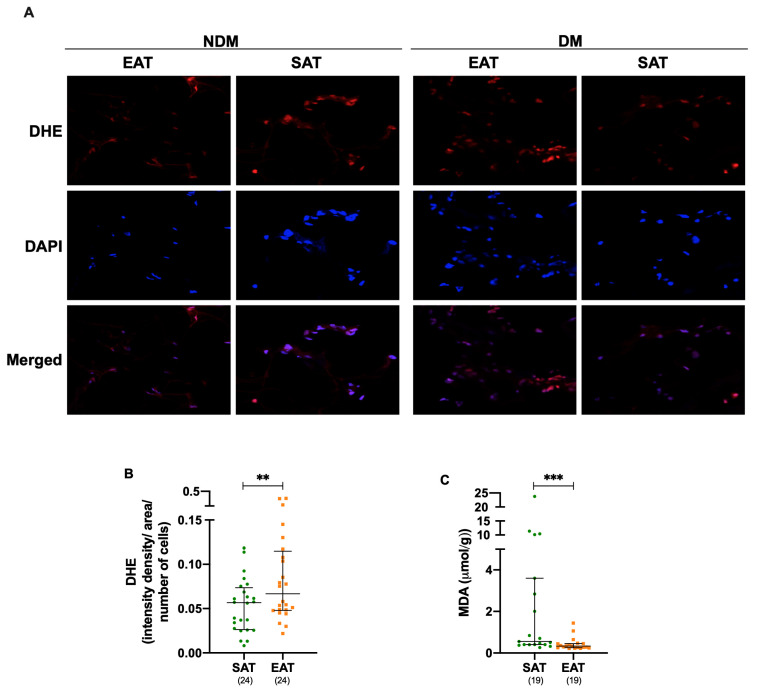
Oxidative stress is elevated in epicardial adipose tissue. DHE oxidation (**A**,**B**) was assessed by microscopy, and the integrated density per field area was quantified and normalized to the number of cells within the same field area. MDA levels were measured by high-performance liquid chromatography (HPLC) (**C**). The number of patients included in each assay is indicated. A *p* ≤ 0.05 was considered statistically different, ** *p* ≤ 0.01; *** *p* ≤ 0.001. DAPI, 4′,6-diamidino-2-phenylindole; DHE, dihydroethidium; EAT, epicardial adipose tissue; MDA, malondialdehyde; SAT, subcutaneous adipose tissue.

Assessment of ROS markers and redox mechanisms is first described in EAT and SAT across all the participants, without stratifying for DM or CAD. DHE fluorescence analysis revealed a significant increase in ROS levels in EAT compared to SAT (DHE, *p* = 0.004), as shown in Figure 2A,B and Table S4. In contrast, lipid peroxidation levels were significantly decreased in EAT than in SAT (MDA, *p* ≤ 0.001), as seen in Figure 2C and Table S4.

Next, we investigated the impact of DM and CAD on the observed differences in ROS and MDA between EAT and SAT. An increase in the DHE fluorescence was detected in EAT from both the NDM (*p* = 0.045) and CAD (*p* = 0.010) groups and shown in Table S5. Meanwhile, MDA levels were consistently decreased in EAT compared to the respective SAT across all groups, except for CAD: NDM (*p* = 0.048), DM (*p* = 0.004), and NCAD (*p* = 0.005), as shown in Table S5.

No differences in ROS or MDA levels were observed in EAT when comparing the presence of DM (EAT NDM versus EAT DM) or CAD (EAT NCAD versus EAT CAD), as shown in Table S5.

### 3.4. Antioxidant Defenses Are Compromised in Epicardial Adipose Tissue of Patients Elected for Cardiac Surgery

**Figure 3:**
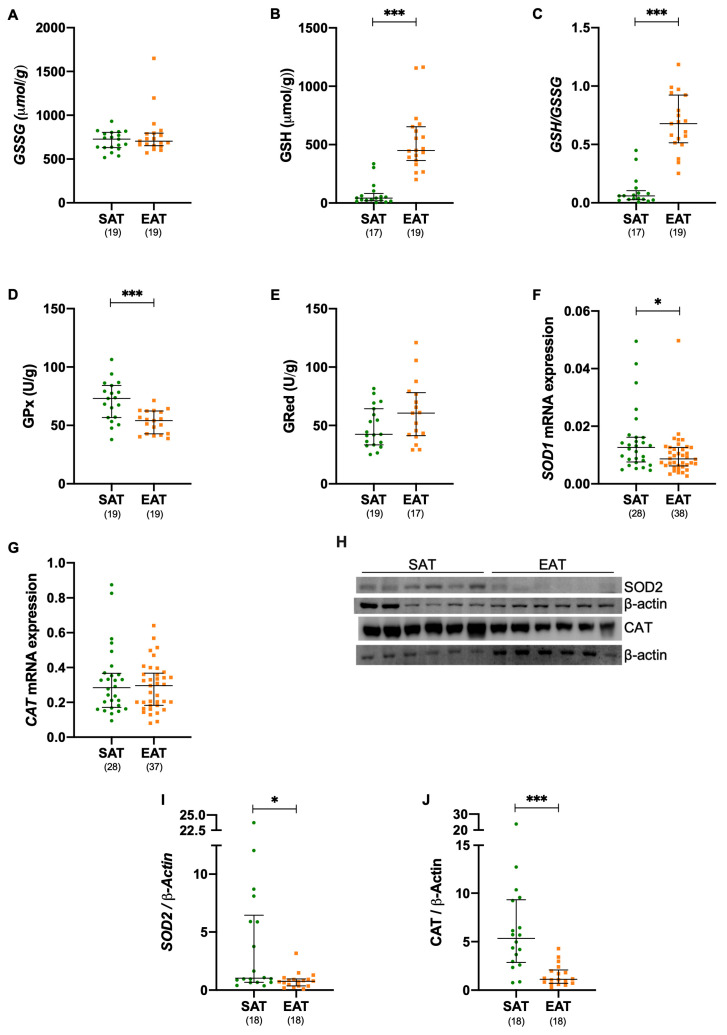
Antioxidant defenses are compromised in epicardial adipose tissue. Oxidized glutathione (GSSG, **A**) and reduced glutathione (GSH, **B**) levels were quantified and expressed as µmol/g protein, with the GSH/GSSG ratio calculated accordingly (**C**). Glutathione peroxidase (GPx, **D**) and glutathione reductase (GRed, **E**) activities were determined spectrophotometrically and expressed as U/g protein. SOD1 (**F**) and CAT (**G**) mRNA expression levels were analyzed by RT-qPCR, while SOD2 (**H**,**I**) and CAT (**H**,**J**) protein levels were quantified by Western blot. Gene and protein expression levels were normalized to the housekeeping gene or protein β-actin, respectively. The number of patients included in each assay is indicated. A *p* ≤ 0.05 was considered statistically different, * *p* ≤ 0.05; *** *p* ≤ 0.001. CAT, catalase; EAT, epicardial adipose tissue; GPx, glutathione peroxidase; GRed, glutathione reductase; GSH, reduced glutathione; GSSG, oxidized glutathione; SAT, subcutaneous adipose tissue; SOD, superoxide dismutase.

We evaluated the antioxidant defense system in EAT compared to SAT in all the subjects undergoing cardiac surgery, without first stratifying for DM or CAD. The glutathione (GSH), a key ROS scavenger, exists in either its reduced (GSH) form, which neutralizes ROS, or in its oxidized (GSSG) form. No differences were observed in GSSG levels between EAT and SAT, as seen in Figure 3A and Table S6. However, GSH levels were significantly elevated (*p* ≤ 0.001) in EAT, as shown in Figure 3B and Table S6, leading to a substantial increase in the intracellular redox buffer ratio GSH/GSSG (*p* ≤ 0.001), in EAT relative to SAT, as seen in Figure 3C and Table S6. Additionally, GPx activity (*p* ≤ 0.001) was significantly reduced in EAT compared to SAT, as shown in Figure 2D and Table S6. Despite an increase of 43% in GRed activity in EAT, this difference did not reach statistical significance, as seen in Figure 2E and Table S6. Furthermore, both SOD1 gene expression (*p* = 0.026), as seen in Figure 3F and Table S6, and mitochondrial SOD2 protein level (*p* = 0.001), as presented in Figure 3H,I and Table S6, were reduced in EAT. While CAT gene expression levels remained unchanged between EAT and SAT, as shown in Figure 3G and Table S6, CAT protein levels were significantly reduced (*p* ≤ 0.001) in EAT, as seen in Figure 3H,J and Table S6.

When evaluating the impact of DM and CAD, GSH levels and the corresponding intracellular redox buffer ratio (GSH/GSSG) were significantly increased in EAT, in the NDM (*p* = 0.004), DM (*p* = 0.008), NCAD (*p* ≤ 0.001), and CAD *p* ≤ 0.001), as seen in Table S7. Interestingly, while GPx activity was reduced in EAT from all groups, this difference reached statical significance in the NDM (*p* = 0.037), DM (*p* = 0.039), and NCAD (*p* = 0.005), as seen in Table S7. In addition, CAT protein levels were significantly decreased in EAT for patients with DM (*p* ≤ 0.001), and CAD (*p* ≤ 0.001), when compared to their respective SAT, as shown in Table S7. Moreover, while no significant differences in ROS scavengers were observed in EAT when evaluating the presence of DM, CAD was associated with a significant reduction in both SOD2 (*p* = 0.035) and CAT (*p* = 0.001) protein levels in EAT from patients with CAD compared with those in the NCAD group, as seen in Table S7.

## 4. Discussion

Among the various forms of CVD, CAD is one of the most prevalent, accounting for a significant proportion of the CVD-related deaths [1]. Additionally, CVD is also the primary cause of death among diabetic patients [23]. Adipose tissue remodeling, insulin resistance, and inflammation are important hallmarks not only for DM, but also for CAD development [24,25]. Despite being one of the least studied human visceral adipose tissues, EAT holds a unique and critical role in cardiac health. EAT serves local essential functions, including nutritional, energetic, and thermogenic regulation, while maintaining crosstalk with neighboring cardiac cells [5,6,7,8,9]. In previous studies we have demonstrated that EAT exhibits impaired glucose uptake and lipid metabolism [9], as well as increased unfolded protein response (UPR) and autophagy, when compared to SAT [8], from patients undergoing cardiac surgery. Therefore, this study aims to go deeper and reveal important key differences in mitochondrial fusion and fission proteins markers, as well as oxidative stress mechanisms within EAT compared to SAT. Additionally, it evaluated the potential impact of DM and CAD on these processes.

Overall, mitochondrial dynamics and antioxidant defenses were significantly altered in EAT compared to SAT. Interestingly, when patients were subsequently stratified into groups based on DM or CAD, these initial tissue-specific alterations largely persisted across the group types (NDM, DM; NCAD, or CAD). The key findings suggest that mitochondrial dynamics and antioxidant defense alterations are primarily tissue-dependent rather than disease specific. In patients with advanced cardiac disease, such as CAD and regardless of DM status, some degree of insulin resistance is commonly observed, mirroring certain DM-related outcomes [8,26,27]. Notably, a previous study with a similar cohort has shown no significant differences in the homeostasis model assessment–insulin resistance (HOMA-IR) index, between patients with and without DM undergoing cardiac surgery [8]. Additionally, over half of the patients with DM took antidiabetic medication, which, together with their prescribed cardiac treatment, may have masked potential differences, when comparing disease status [8,26,27].

Mitochondria fission and fusion cycles, referred to as ‘mitochondrial dynamics’, maintain mitochondrial shape, distribution, and size [28]. These mechanisms are responsible for tissue homeostasis by enhancing energy production, cell survival, and cell proliferation [29]. Myocardium mitochondria may alter its morphology through fusion and fission events to respond to the changing energy demand of heart cells [30]. Interestingly, the expression of either fusion and fission mitochondrial dynamics genes was increased in EAT compared to SAT. The higher expression of both fusion and fission markers has been related with better mitochondria viability, stability, and function [11,29,30,31,32]. Moreover, the expression of *DRP1* in EAT was twice that of SAT. *DRP1* facilitates mitochondrial quality control by mitophagy [11,30].

The transcriptional coactivator PGC-1α controls important aspects of mitochondrial biogenesis through the up-regulation of nuclear- and mitochondrial-encoded gene expression [33]. It positively regulates glucose transporter protein 4 (GLUT4) expression and favors increased glucose uptake [33]. In fact, in a similar cohort, both glucose uptake and *GLUT4* mRNA levels were impaired in the EAT [9]. A study conducted by Sacks and colleagues described that gene expression of brown adipose tissue (BAT) markers, including PARGC1A, were significantly elevated in the EAT of adult patients with cardiac disease, when compared to SAT [6]. However, we observed a significant decrease in PGC-1α protein content in EAT, with no changes in gene expression.

Intracellular ROS represents an important mediator of mitochondrial stress signaling to promote cellular adaptations [34]. A previous study has identified a direct correlation between ROS production in EAT, from patients with CAD, and the prevalence of impaired insulin action, glycemia management, and inflammation [35]. In fact, ROS over-production may increase atherosclerosis, myocardial infarction, heart failure, and atrial fibrillation [36,37,38], as well as other important aspects evidenced in chronic diseases, like DM, obesity, and aging [39].

In the present study, ROS production in EAT was evaluated directly through superoxide quantification by DHE oxidation, and the presence of lipid peroxidation products by MDA quantification. An increase in DHE in EAT was observed when compared to SAT. Interestingly, this was mainly supported by increased ROS in EAT from patients without DM and CAD, when compared to SAT, since DHE measurements remained similar in tissues from patients with DM and in the NCAD group, likely driven by the medication DM patients are taking. In patients with obesity and types 2 DM, MDA accumulation has been observed in isolated mitochondria, when compared to controls (without obesity or diabetes) [40]. However, in the present study, EAT presented lower MDA levels when compared with SAT. Importantly, EAT has been described as a buffer to prevent cardiomyocyte lipotoxicity [41].

The antioxidant defense system neutralizes the adverse effects of ROS in the mitochondria and in the cytoplasm [42]. Reduction in antioxidant enzyme activities such as glutathione, SOD, and catalase has been observed in adipose tissue isolated mitochondria from individuals with obesity and DM [40,42]. Similarly, reduced GPx activity was observed in EAT when compared with SAT, indicating the accumulation of GSH and ROS. Glutathione is in fact one of the most powerful antioxidants in the cardiovascular system [43].

Moreover, catalase is the most abundant peroxisomal antioxidant enzyme [44]. On the other hand, there are several SOD forms, acting at different locations. In humans, SOD1 plays an important role in the mitochondrial membranal space and in the cytoplasm, while SOD2 is in the mitochondria matrix, and SOD3 is in the extracellular fluid [44]. To note that, since *SOD1* transcripts and both SOD2 and catalase were decreased in EAT when compared to SAT, the observed ROS over-production could perhaps be ascribed not only to the impaired respiratory chain function, but also to the reduced oxidative defense system, as previously described [45].

Moreover, catalase and SOD2 were the only antioxidant defense markers significantly reduced in the EAT of patients with CAD compared with those in the NCAD group. Notably, previous studies have described that moderate cardiac overexpression of SOD2 and/or catalase may lead to a decrease in intracellular ROS, thus ameliorating mitochondrial function and other ultrastructural defects, in turn minimizing myocardial degeneration, and leading to a significant improvement in cardiac function [46].

Moreover, previous reports identified that DRP1 and ROS have a reciprocal relationship, in which ROS improves DRP1 activation, and vice versa [47]. Although the underlying mechanisms remain elusive, increases in DRP1 and mitochondrial fragmentation are interlinked with ROS production [29]. This mutual increase in DRP1 and ROS could be key players, generating mitochondrial dysfunction and, in turn contributing to insulin resistance development, as observed in EAT [9].

This study has several limitations that deserve consideration. First, we lacked access to truly healthy control subjects for adipose tissue biopsies, as EAT can only be ethically collected during cardiac surgeries. Therefore, tissues from patients in the NDM or NCAD groups were used as internal references, but their underlying cardiac indications likely reflect subclinical pathologies, limiting the interpretation of baseline metabolic profiles. Second, most patients were not medication-naïve. Common therapies, such as antidiabetic and cardiovascular drugs, may influence adipose tissue metabolism [48,49,50].

While these treatments enhance the translation relevance of our findings, they may obscure disease-specific metabolic differences. Similar tissues might be collected post-mortem from relatively healthy patients, but factors such as time of death and delay in tissue harvesting can compromise sample quality. Third, although we evaluated the independent effects of DM and CAD, we were unable to perform a fully factorial four-group analysis (NDM NCAD, NDM CAD, DM NCAD, and DM CAD) due to biopsy material constraints and group size imbalances. To address this, we consistently prioritized intra-patient comparisons using paired EAT and SAT samples whenever technically possible, thereby minimizing inter-subject variability. Finally, we applied non-parametric tests, Wilcoxon signed-rank tests for intra-individual (paired samples) comparisons and Mann–Whitney U tests for inter-group comparisons (DM or CAD status), which are well suited for a small sample size and non-normally distributed data. While alternative approaches, such as Kruskal–Wallis with post hoc correction or multi-variable regression models, could provide complementary insights in larger, more balanced cohorts, our selected approach was appropriate and statistically robust for the structure and scope of this study.

## 5. Conclusions

This study provides novel mechanistic insights into the mitochondrial and redox profile of EAT, revealing a distinct tissue that is metabolically dynamic and with a different phenotype compared to SAT. The upregulation of both mitochondrial fusion and fission markers in EAT suggests active mitochondrial remodeling, likely reflecting an adaptative response to persistent cellular stress. Notably, although EAT exhibited elevated ROS levels and reduced antioxidant enzyme expression, it showed lower lipid peroxidation, along with an increased mitochondrial respiration and stable mitochondrial-to-nuclear DNA (mtDNA:nDNA) ratio compared to SAT. These fundings suggest that EAT maintains functional resilience through coordinated stress-adaptive mechanisms.

Importantly, this work builds upon our previous efforts to characterize EAT biology, contributing to a broader understating of its metabolic and functional specificity [8,9]. Due to the limited amount of tissue available from each EAT biopsy, it was not feasible to perform all planned analyses on a single sample. To address this limitation, we increased the number of participants and allocated the available samples into separate experimental groups, each focused on specific metabolic pathways. This study resembles a targeted approach to investigate key proteins involved in mitochondrial dynamics and oxidative stress, providing mechanistic insights with high sensitivity and reproducibility, particularly valuable given the limited biopsy material. The future application of broader, unbiased methods such as proteomic analyses could further enrich the understanding of EAT biology by uncovering additional pathways and molecular alterations beyond the scope of the present study.

Methodologically, this study is the first to ingrate the analysis of mitochondrial dynamics and oxidative stress in EAT while also stratifying findings by relevant clinical comorbidities (DM and CAD). Overall, our findings suggest that EAT exists in a highly stressed state yet engages in a compensatory mechanism that preserves the local function despite metabolic and oxidative challenges. These insights underscore the significance of EAT as an active player in cardiometabolic disease and highlight the importance of tissue-specific molecular profiling in understanding its complex role.

## Figures and Tables

**Table 1:** Primer sequences.

Name	Sequence (5′-3′)	Ta (°C)
β-actin	Forward: AACTACCTTCAACTCCATC Reverse: TGATCTTGATCTTCATTGTG	60
CAT	Forward: AACTTCACTGAGGTCCAC Reverse: ATCGCATTCTTAGGCTTCT	60
DRP1	Forward: AAGAAGAGTGTAACTGATT Reverse: AGAAGAGACTGATACTGA	52
FIS1	Forward: CAATGATGACATCCGTAA Reverse: AGGTAGAAGACGTAATCC	52
MFN1	Forward: ATAATGGCAGAACCTGTT Reverse: GGATTCTTATATGTTGCTTCA	55
MFN2	Forward: CAGAAGAGAACTCAGAATC Reverse: CTTGACTGTGACGATAGA	52
OPA1	Forward: TGTATTCTGAAGTTCTTGATGT Reverse: ATCTCCAACCACAACAAC	55
PGC1A	Forward: GAGGAATATCAGCACGAGAGG Reverse: ACTTCAAAACGGTCCCTCAG	60
SOD1	Forward: ATGGCCCAATAAACATTC Reverse: CTATACAAATCTTCCAAGTGA	60

CAT, Catalase; DRP1, Dynamin-1-like protein; FIS1, Fission 1; MFN1, Mitofusin 1; MFN2, Mitofusin 2; OPA1, Optic atrophy 1; PGC1A, Peroxisome proliferator-activated receptor-gamma coactivator 1 alpha; SOD1, Superoxide dismutase; Ta, annealing temperature.

**Table 2:** Demographic and clinical characteristics of the study population (*n* = 128).

	NDM	DM	*p*-Value ^a^	NCAD	CAD	*p*-Value ^b^
N	68	60		67	61	
Male (M)	55 (81%)	44 (73%)	0.31	44 (66%)	55 (90%)	**0.001**
Age (years)	67.0 (60.0–76.0)	71.5 (65.0–76.8)	0.052	71.0 (63.0–77.0)	68.0 (62.0–74.0)	0.17
**Cardiovascular risk factors**						
Diabetes Mellitus				31 (46%)	29 (49%)	0.88
Hypertension	43 (63%)	49 (82%)	**0.021**	42 (63%)	45 (74%)	**0.015**
Dyslipidemia	47 (69%)	46 (77%)	0.34	40 (60%)	53 (87%)	≤0.001
**Smoking**						
Nonsmoker	45 (66%)	39 (65%)	0.89	50 (75%)	34 (56%)	**0.025**
Ex-smoker	21 (35%)	16 (27%)	0.60	16 (24%)	22 (34%)	0.08
Recent smoker history	0 (0%)	2 (3%)	0.13	0 (0%)	2 (3%)	0.14
Active smoker	2 (3%)	3 (5%)	0.54	2 (3%)	3 (2%)	0.71
BMI	26.52 ± 0.35	27.31 ± 0.29	0.030	27.0 (25.0–29.0)	28.0 (26.0–29.0)	0.23
Family history of heart disease	8 (12%)	7 (12%)	0.97	1 (2%)	14 (23%)	**≤0.001**
**Medication**						
Antiplatelet	35 (51%)	37 (62%)	0.25	22 (33%)	50 (82%)	**≤0.001**
Antiarrhythmic	6 (9%)	10 (17%)	0.18	14 (21%)	4 (7%)	0.052
Anticoagulant	10 (15%)	11 (18%)	0.55	13 (14%)	8 (13%)	0.39
Insulin	0 (0%)	11 (18%)	**≤0.001**	4 (6%)	7 (11%)	0.27
**Oral antidiabetic**						
Biguanide	0 (0%)	20 (33%)	**≤0.001**	15 (21%)	5 (8%)	**0.027**
DPP4 inhibitor	0 (0%)	9 (15%)	**≤0.001**	6 (9%)	3 (5%)	0.37
DPP4 inhibitor + Biguanide	0 (0%)	14 (23%)	**≤0.001**	6 (9%)	8 (13%)	0.45
Sulfonylurea	0 (0%)	15 (25%)	**≤0.001**	7 (10%)	8 (13%)	0.64
Diuretic	34 (50%)	30 (50%)	>0.99	37 (55%)	27 (44%)	0.22
ACEI	22 (32%)	25 (22%)	0.28	24 (36%)	23 (38%)	0.83
ARB	16 (24%)	13 (22%)	0.84	15 (22%)	14 (23%)	0.98
β blocker	45 (66%)	30 (50%)	0.10	32 (47%)	43 (70%)	**0.015**
Calcium channel blocker	9 (13%)	10 (17%)	0.57	5 (8%)	14 (23%)	**0.014**
Electrolyte—KCl	8 (12%)	8 (13%)	0.79	10 (15%)	6 (10%)	0.39
Statins	41 (60%)	49 (82%)	**0.008**	38 (57%)	52 (85%)	**≤0.001**
Vasodilator	13 (19%)	13 (22%)	0.72	7 (10%)	19 (31%)	**0.004**

Quantitative measurements are presented as means ± SEM. For categorical variables, a χ^2^ test was applied. For normally distributed data, a parametric t-test was performed, whereas a nonparametric Mann–Whitney test was applied for non-normally distributed data. Significant *p*-values (*p* ≤ 0.05) are highlighted in bold. ACEI, angiotensin-converting enzyme inhibitor; ARBs, angiotensin II receptor blockers; BMI, body mass index; CAD, coronary artery disease group; DM, diabetes mellitus group; DPP-4, dipeptidyl peptidase-4; KCl, potassium chloride; NCAD, non-coronary artery disease group; NDM, non-diabetes mellitus group; ^a^ NDM versus DM; ^b^ NCAD versus CAD.

## Data Availability

The data that support the findings of this study are available from the corresponding author upon reasonable request.

## Supplementary Materials

The following supporting information can be downloaded at: https://www.mdpi.com/article/10.3390/metabo15070481/s1, Table S1: Anthropometric and Clinical characteristics of the study population according to cardiac disease (*n* = 128); Table S2: Mitochondrial biogenesis gene expression in EAT and SAT from patients subjected to cardiac surgery; Table S3: The influence of DM and CAD in the Mitochondrial biogenesis gene expression in EAT and SAT from patients subjected to cardiac surgery; Table S4: Reactive oxygen species accumulation in EAT and SAT from patients subjected to cardiac surgery; Table S5: The influence of DM and CAD in the EAT and SAT reactive oxygen species accumulation from patients subjected to cardiac surgery; Table S6: Expression levels and activity of the antioxidant defense mechanisms in EAT and SAT from patients subjected to cardiac surgery; Table S7: The influence of DM and CAD in the expression levels and activity of the antioxidant defense mechanisms in EAT and SAT from patients subjected to cardiac surgery.

## Abbreviations

The following abbreviations are used in this manuscript:

ACEI	Angiotensin-converting enzyme inhibitor
ARB	Angiotensin II receptor blocker
BMI	Body mass index
BAT	Brown adipose tissue
CAT	Catalase
CABG	Coronary artery bypass grafting
CAD	Coronary artery disease
CVD	Cardiovascular disease
DHE	Dihydriethidine
DM	Diabetic
DPP-4	Dipeptidyl peptidase-4
DRP1	Dynamin-1-like protein
EAT	Epicardial adipose tissue
FIS1	Fission protein 1
GLUT4	Glucose transporter proteins 4
GPx	Glutathione peroxidase
GRed	Glutathione reductase
GSSG	Oxidized glutathione
GSH	Reduced glutathione
MDA	Malondialdehyde
MNF1	Mitofusin 1
MNF2	Mitofusin 2
OCT	Optimal cutting temperature
OPA1	Optic atrophy 1
PGC1a	Peroxisome proliferator-activated receptor-gamma coactivator 1 alpha
ROS	Reactive oxygen species
SAT	Subcutaneous adipose tissue
SOD	Superoxide dismutase
Ta	Annealing temperature
UPR	Unfold protein response
