# Supplementary material for: Enhanced Mitochondrial Dynamics and Reactive Oxygen Species Levels with Reduced Antioxidant Defenses in Human Epicardial Adipose Tissue
Source: Metabolites. 2025 Jul 16;15(7):481. doi: 10.3390/metabo15070481 (PMC12300022; doi:10.3390/metabo15070481)
Supplement: Supplementary file 1 [file metabolites-15-00481-s001.zip › metabolites-3715746-supplementary.pdf]

metabolites

Article

Enhanced Mitochondrial Dynamics and Reactive Oxygen Species Levels with Reduced Antioxidant Defenses in Human Epicardial Adipose Tissue

Ana Burgeiro [1,2,3,†], Diana Santos [1,2,4,5,*,†], Ana Catarina R. G. Fonseca [1,2,†], Inês Baldeiras [1,2], Ermelindo C. Leal [1,2,5], João Moura [1,2], João Costa-Nunes [1,2], Patrícia Monteiro Seraphim [1,2,6], Aryane Oliveira [1,2], António Canotilho [7], Gonçalo Coutinho [7], David Prieto [7], Pedro Antunes [7], Manuel Antunes [8] and Eugenia Carvalho [1,2,5,*]

[1] CNC—Center for Neuroscience and Cell Biology, University of Coimbra, 3004-504 Coimbra, Portugal; burgeiroana@gmail.com (A.B.); anacatarinafonseca@hotmail.com (A.C.R.G.F.); ines.baldeiras@sapo.pt (I.B.); ecleal@cnc.uc.pt (E.C.L.); jmouraalves@gmail.com (J.M.); jpcosta.nunes@gmail.com (J.C.-N.); pm.seraphim@unesp.br (P.M.S.); aryanecruz.op@gmail.com (A.O.)
[2] CiBB—Center for Innovative Biomedicine and Biotechnology, University of Coimbra, 3004-504 Coimbra, Portugal
[3] Local Health Unit of the Leiria Region, E.P.E, 2140-197 Leiria, Portugal
[4] PhD Programme in Experimental Biology and Biomedicine (PDBEB), Institute for Interdisciplinary Research, University of Coimbra, 3030-789 Coimbra, Portugal
[5] Institute for Interdisciplinary Research, University of Coimbra, 3030-789 Coimbra, Portugal
[6] Department of Physiotherapy, School of Sciences and Technology, Campus Presidente Prudente, Sao Paulo State University (UNESP), Sao Paulo 19060-900, Brazil
[7] Cardiothoracic Surgery Unit, University Hospital of Coimbra, 3000-075 Coimbra, Portugal; ajcano@sapo.pt (A.C.); goncalofcoutinho@gmail.com (G.C.); dprietodelaplaza@gmail.com (D.P.); p.engracia.antunes@gmail.com (P.A.)
[8] University Clinic for Cardiothoracic Surgery, Faculty of Medicine, University Hospital of Coimbra, 3000-548 Coimbra, Portugal; mjantunes48@sapo.pt
* Correspondence: dfsantos@cnc.uc.pt (D.S.); ecarvalh@cnc.uc.pt (E.C.)
† These authors contributed equally to this work.

 https://doi.org/10.3390/metabo15070481

Supplementary Materials

Table S1. Anthropometric and clinical characteristics of the study population according to cardiac disease ($n = 128$).

**NCAD Patients ($n = 67$)**	**NDM**	**DM**	**p-Value**
N	36	31	
Male (M)	26 (72%)	18 (58%)	0.22
Age (years)	67.0 (59.25–76.00)	73.0 (69.0–78.0)	0.052
BMI	26.35 ± 0.52	26.91 ± 0.47	0.42
**Dysfunctional valve**			
Aortic	24 (67%)	25 (80%)	0.20
Mitral	8 (25%)	2 (6%)	0.3
Tricuspid	4 (11%)	0 (0%)	0.06
Bivalvular	0 (0%)	4 (13%)	0.026
**CAD Patients ($n = 61$)**	**NDM**	**DM**	**p-Value**
N	32	29	
Male (M)	29 (91%)	25 (86%)	0.90
Age (years)	62.2 ± 1.7	67.7± 1.5	0.52
BMI	27.7 (24.3–28.9)	28.0 (26.0–29.0)	0.23
**Dysfunctional valve**			
Aortic	8 (25%)	7 (24%)	0.44
Mitral	2 (6%)	1 (3%)	0.83
Tricuspid	1 (3%)	0 (0%)	0.41
Bivalvular	1 (3%)	0 (0%)	0.41
**Number of revascularized cardiac vessels**			
1 vessel disease	6 (19%)	8 (28%)	0.42
2 vessels disease	11 (34%)	9 (31%)	0.85
3 vessels disease	15 (47%)	12 (41%)	0.76

Quantitative measurements are presented as means ± SEM. For categorical variables, a χ^2 test was applied. For normally distributed data, a parametric t-test was performed, whereas a nonparametric Mann–Whitney test was applied for non-normally distributed data. Significant *p*-values ($p \leq 0.05$) are highlighted in bold. NDM, non-diabetic group; DM, Diabetic group; NCAD, Non-coronary artery disease group; CAD, coronary artery disease group; BMI, body mass index; DPP-4, dipeptidyl peptidase-4; ACEI, angiotensin-converting enzyme inhibitor; ARBs, angiotensin II receptor blockers.

Table S2. Mitochondrial biogenesis gene expression in EAT and SAT from patients subjected to cardiac surgery.

	SAT	*n*	EAT	*n*	p-Value
PCG1-α/β-actin	2.36 (1.47–3.69)	24	1.68 (1.43–2.33)	24	**0.015**
PPARGC1A mRNA expression	0.0007 (0.0004–0.0013)	28	0.0008 (0.0005–0.0010)	28	0.56
MFN1 mRNA expression	0.021 (0.018–0.026)	31	0.030 (0.024–0.041)	38	≤0.001
MFN2 mRNA expression	0.0132 (0.010–0.015)	28	0.016 (0.012–0.020)	38	0.038
OPA1 mRNA expression	0.029 (0.024–0.035)	28	0.046 (0.033–0.067)	33	≤0.001
DRP1 mRNA expression	0.013 (0.012–0.017)	31	0.027 (0.020–0.033)	38	≤0.001
FIS1 mRNA expressio*n*	0.018 (0.016 –0.024)	28	0.032 (0.021–0.048)	37	≤0.001

Quantitative measurements are presented as median (interquartile range). In accordance with the data set a non-parametric Wilcoxon paired test or a Mann–Whitney test was applied. Significant *p*-values (p ≤ 0.05) are highlighted in bold. The number of patients included in each assay is indicated. PGC1A/*PPARGC1A*, Peroxisome proliferator-activated receptor-gamma coactivator 1alpha; MFN1, Mitofusin 1; MFN2, Mitofusin 2; OPA1, Optic atrophy 1; DRP1, Dynamin-1-like protein; FIS1, Fission 1; SAT, subcutaneous adipose tissue; EAT, epicardial adipose tissue.

Table S3. The influence of DM and CAD in the Mitochondrial biogenesis gene expression in EAT and SAT from patients subjected to cardiac surgery.

	SAT NDM	n	EAT NDM	n	*p*-Value [a]	SAT DM	n	EAT DM	n	p-Value [b]	p-Value [c]
PCG1-α/β-actin	2.04 (1.32–2.53)	12	1.54 (1.43–2.88)	12	0.30	3.52 (1.57–5.00)	12	1.88 (135–2.33)	12	**0.016**	0.93
PPARGC1A mRNA expression	0.0006 (0.0004–0.0010	13	0.0008 (0.0004–0.0009)	13	0.98	0.0007 (0.0004–0.0013)	15	0.0007 (0.0005–0.0010)	15	0.43	0.88
MFN mRNA expression	0.023 (0.019–0.026)	19	0.030 (0.022–0.041)	**22**	**0.011**	0.018 (0.016–0.022)	12	0.031 (0.027–0.040)	16	**≤0.001**	0.59
MFN2 mRNA expression	0.013 (0.011–0.015)	17	0.016 (0.011–0.018)	22	0.17	0.011 (0.009–0.018)	11	0.015 (0.012–0.019)	16	0.15	0.71
OPA1 mRNA expression	0.029 (0.027–0.034)	17	0.047 (0.033–0.070)	**19**	**0.017**	0.028 (0.023–0.0365)	11	0.043 (0.036–0.055)	14	**0.013**	0.85
DRP1 mRNA expression	0.013 (0.012–0.017)	19	0.025 (0.020–0.031)	**22**	**≤0.001**	0.013 (0.011–0.016)	12	0.028 (0.020–0.038)	16	**≤0.001**	0.25
FIS1 mRNA expression	0.018 (0.017 –0.024)	17	0.032 (0.019–0.050)	**22**	**0.013**	0.018 (0.013–0.026)	11	0.032 (0.021–0.045)	15	**0.040**	0.99
	**SAT NCAD**	n	**EAT NCAD**	n	*p*-Value [d]	**SAT CAD**	n	**EAT CAD**	n	p-Value [e]	p-Value [f]
PCG1-α/β-actin	3.45 (1.30–5.64)	13	1.86 (1.36–4.10)	13	0.08	2.053 (1.68–2.51)	11	1.48 (1.43–2.17)	11	0.10	0.39
PPARGC1A mRNA expression	0.0005 (0.0005–0.0016)	9	0.0008 (0.0004–0.0012)	9	0.48	0.0007 (0.0004–0.0013)	19	0.0007 (0.0005–0.0009)	19	0.93	0.67
MFN1 mRNA expression	0.021 (0.018–0.024)	12	0.030 (0.026–0.048)	13	**≤0.001**	0.022 (0.017–0.019)	19	0.030 (0.024–0.039)	25	**0.017**	0.62
MFN2 mRNAexpression	0.012 (0.010–0.015)	11	0.017 (0.012–0.026)	13	**0.004**	0.014 (0.010–0.018)	17	0.015 (0.011–0.019)	25	0.58	0.22
OPA1 mRNA expression	0.029 (0.022–0.035)	11	0.043 (0.034–0.077)	13	**0.002**	0.028 (0.026–0.038)	17	0.046 (0.031 — 0.055)	20	0.08	0.54
DRP1 mRNA expression	0.012 (0.012–0.014)	12	0.027 (0.021–0.035)	13	**≤0.001**	0.015 (0.011–0.018)	19	0.027 (0.020–0.032)	25	**≤0.001**	0.81
FIS mRNA expression	0.018 (0.017–0.023)	11	0.035 (0.027 –0.057)	13	**≤0.001**	0.018 (0.015–0.029)	17	0.031 (0.016–0.045)	24	0.11	0.19

Quantitative measurements are presented as median (interquartile range). In accordance with the data set a non-parametric Wilcoxon paired test or a Mann–Whitney test was applied for the non-normally distributed data. Significant *p*-values (p ≤ 0.05) are highlighted in bold. The number of patients included in each assay is indicated. PGC1A / *PPARGC1A*, Peroxisome proliferator-activated receptor-gamma coactivator 1alpha; MFN1, Mitofusin 1; MFN2, Mitofusin 2; OPA1, Optic atrophy 1; DRP1, Dynamin-1-like protein; FIS1, Fission 1; NDM, Non-Diabetic group; DM, Diabetic

group; NCAD, Non-coronary artery disease group; CAD, coronary artery disease group; SAT, subcutaneous adipose tissue; EAT, epicardial adipose tissue. [a] SAT NDM versus EAT NDM; [b] SAT DM versus EAT DM; [c] EAT NDM versus EAT DM; [d] SAT NCAD versus EAT CAD; [e] SAT CAD versus EAT CAD; [f] EAT NCAD versus EAT CAD.

Table S4. Reactive oxygen species accumulation in EAT and SAT from patients subjected to cardiac surgery.

	SAT	n	EAT	n	p-Value
DHE	0.06 (0.03–0.07)	24	0.07 (0.05–0.11)	24	**0.004**
MDA (μmol/g)	0.55 (0.40–3.60)	19	0.33 (0.26–0.45)	19	**≤0.001**

Quantitative measurements are presented as median (interquartile range). In accordance with the data set a non-parametric Wilcoxon paired test or a Mann–Whitney test was applied. Significant *p*-values (p ≤ 0.05) are highlighted in bold. The number of patients included in each assay is indicated. DHE, dihydriethidine; MDA, malondialdehyde; SAT, subcutaneous adipose tissue; EAT, epicardial adipose tissue.

Table S5. The influence of DM and CAD in the EAT and SAT reactive oxygen species accumulation from subjected to cardiac surgery.

	SAT NDM	n	EAT NDM	n	p-Value [a]	SAT NDM	n	EAT DM	n	p-Value [b]	p-Value [b]
DHE	0.04 (0.02–0.07)	13	0.06 (0.05–0.10)	16	**0.045**	0.06 (0.05–0.11)	11	0.05 (0.04–0.51)	8	0.78	0.74
MDA (μmol/g)	0.49 (0.38–10.64)	10	0.39 (0.28–0.75)	10	**0.048**	0.84 (0.40–3.22)	9	0.27 (0.24–0.38)	9	**0.004**	0.13
	**SAT NCAD**	**n**	**EAT NCAD**	**n**	**p-Value [d]**	**SAT CAD**	**n**	**EAT CAD**	**n**	**p-Value [e]**	**p-Value [f]**
DHE	0.06 (0.04–0.08)	15	0.05 (0.05–0.13)	15	0.60	0.03 (0.02–0.07)	9	0.08 (0.05–0.11)	9	**0.010**	0.41
MDA (μmol/g)	0.71 (0.41–6.86)	13	0.30 (0.24–0.52)	13	**0.005**	0.44 (0.37–6.57)	6	0.39 (0.28–0.46)	6	0.06	0.52

Quantitative measurements are presented as median (interquartile range). In accordance with the data set a non-parametric Wilcoxon paired test or a Mann–Whitney test was applied for the non-normally distributed data. Significant *p*-values (p ≤ 0.05) are highlighted in bold. The number of patients included in each assay is indicated. DHE, dihydriethidine; MDA, malondialdehyde; NDM, Non-Diabetic group; DM, Diabetic group; NCAD, Non-coronary artery disease group; CAD, coronary artery disease group; SAT, subcutaneous adipose tissue; EAT, epicardial adipose tissue. [a] SAT NDM versus EAT NDM; [b] SAT DM versus EAT DM; [c] EAT NDM versus EAT DM; [d] SAT NCAD versus EAT CAD; [e] SAT CAD versus EAT CAD; [f] EAT NCAD versus EAT CAD.

Table S6. Expression levels and activity of the antioxidant defense mechanisms in EAT and SAT from patients subjected to cardiac surgery.

	SAT	n	EAT	n	p-Value
GSSG (μmol/g)	726.83 (631.16–652.91)	19	704.38 (653.24–796.43)	19	0.49
GSH (μmol/g)	41.78 (19.22–82.40)	17	450.39 (364.61–652.91)	19	**≤0.001**
GSSG/GSH	0.06 (0.03–0.11)	17	0.68 (0.51–0.92)	19	**≤0.001**
GPx (U/g)	73.07 (56.73–84.10)	19	54.02 (42.80–62.40)	19	**≤0.001**
GRed (U/g)	42.46 (33.45–64.33)	19	60.62 (41.25–78.11)	17	0.19
SOD1 mRNA expression	0.013 (0.008–0.016)	28	0.009 (0.006–0.013)	38	**0.026**
CAT mRNA expression	0.285 (0.171–0.367)	28	0.296 (0.182–0.367)	37	0.79
SOD2/β-actin	1.03 (0.67–6.45)	18	0.76 (0.36–0.96)	18	**0.024**
CAT/β-actin	5.33 (2.87–9.32)	18	1.13 (0.71–2.08)	18	**≤0.001**

Quantitative measurements are presented as median (interquartile range). In accordance with the data set a non-parametric Wilcoxon paired test or a Mann–Whitney test was applied. Significant *p*-values (*p* ≤ 0.05) are highlighted in bold. The number of patients included in each assay is indicated. GSSG, oxidized glutathione; GSH, reduced glutathione; GPx, glutathione peroxidase; GRed,

glutathione reductase; SOD, Superoxide dismutase; CAT, Catalase; NDM, Non-Diabetic; DM, Diabetic; SAT, subcutaneous adipose tissue; EAT, epicardial adipose tissue.

Table S7. The influence of DM and CAD in the expression levels and activity of the antioxidant defense mechanisms in EAT and SAT from patients subjected to cardiac surgery.

	**SAT NDM**	**n**	**EAT NDM**	**n**	***p*-Value** [a]	**SAT NDM**	**n**	**EAT DM**	**n**	**p-Value** [b]	**p-Value** [c]
GSSG (μmol/g)	688.98 (595.62–814.43)	10	751.90 (677.57–918.54)	10	0.32	726.83 (654.84–787.89)	9	671.18 (626.57–764.14)	9	0.82	0.24
GSH (μmol/g)	26.69 (15.36–72.38)	9	498.61 (339.97–778.64)	10	**0.004**	53.12 (22.72–127.37)	8	450.39 (369.21–615.37)	9	**0.008**	0.84
GSSG/GSH	0.04 (0.02–0.10)	9	0.64 (0.47–0.95)	10	**0.004**	0.07 (0.03–0.16)	8	0.68 (0.55–0.84)	9	**0.008**	0.97
GPx (U/g)	69.45 (49.81–84.78)	10	52.69 (40.85–61.78)	10	**0.037**	73.07 (62.13–82.74)	9	55.04 (45.73–62.55)	9	**0.039**	0.50
GRed (U/g)	41.60 (30.26–46.66)	10	56.52 (40.78–78.69)	8	0.051	59.27 (35.94–74.07)	9	60.62 (38.25–78.89)	9	≥0.99	≥0.99
SOD1 mRNA expression	0.013 (0.008–0.016)	17	0.008 (0.006–0.013)	22	0.06	0.012 (0.008–0.016)	11	0.009 (0.006–0.012)	16	0.23	0.87
CAT mRNA expression	0.331 (0.211–0.409)	17	0.313 (0.160–0.397)	22	0.50	0.211 (0.175–0.285)	11	0.248 (0.202–0.330)	15	0.36	0.94
SOD2/β-actin	0.97 (0.91–6.98)	9	0.70 (0.36–1.10)	9	0.13	1.08 (0.66–8.98)	9	0.82 (0.44–1.00)	9	0.13	0.73
CAT/β-actin	4.36 (1.91–6.28)	9	1.22 (0.99–2.69)	9	0.07	5.72 (3.14–9.96)	9	0.84 (0.59–1.90)	**9**	**≤0.001**	0.28
	**SAT NCAD**	**n**	**EAT NCAD**	**n**	***p*-Value** [d]	**SAT CAD**	***n***	**EAT CAD**	**n**	**p-Value** [e]	**p-Value** [f]
GSSG (μmol/g)	668.81 (516.37–759.91)	13	704.38 (658.65–704.38)	13	0.24	810.92 (611.21–851.69)	6	709.26 (623.92–816.57)	6	0.69	0.77
GSH (μmol/g)	26.69 (15.75–61.83)	11	465.89 (337.15–688.60)	13	**≤0.001**	45.99 (22.68–187.97)	6	412.49 (356.00–592.27)	6	**0.002**	0.52
GSSG/GSH	0.04 (0.02–0.08)	11	0.69 (0.48–0.93)	13	**≤0.001**	0.06 (0.03–0.23)	6	0.56 (0.51–0.80)	6	**0.002**	0.58
GPx (U/g)	78.43 (55.04–86.54)	13	51.36 (42.23–62.55)	13	**0.005**	69.09 (54.74–74.71)	6	54.80 (48.12–58.34)	6	0.09	0.83
GRed (U/g)	44.44 (35.51–61.80)	13	45.52 (33.25–87.81)	11	0.57	39.39 (30.26–69.59)	6	67.79 (49.07–77.52)	6	0.13	0.46
SOD1 mRNA expression	0.010 (0.008–0.014)	11	0.010 (0.006–0.013)	13	0.33	0.013 (0.006–0.021)	17	0.008 (0.006–0.012)	25	0.052	0.55
CAT mRNA expression	0.221 (0.182–0.370)	11	0.294 (0.201–0.452)	12	≥0.99	0.291 (0.165–0.381)	17	0.296 (0.173–0.360)	25	≥0.99	0.47
SOD2/β-actin	5.90 (0.97–12.07)	7	0.93 (0.85–1.30)	7	0.07	0.87 (0.65–1.63)	11	0.65 (0.36–0.79)	11	0.15	**0.035**
CAT/β-actin	6.43 (0.87–10.35)	7	1.97 (0.65–2.97)	7	0.08	4.36 (2.96–6.13)	11	1.06 (0.73–1.22)	11	**≤0.001**	**0.001**

Quantitative measurements are presented as median (interquartile range). In accordance with the data set a non-parametric Wilcoxon paired test or a Mann–Whitney test was applied. Significant *p*-values ($p \leq 0.05$) are highlighted in bold. The number of patients included in each assay is indicated. GSSG, oxidized glutathione; GSH, reduced glutathione; GPx, glutathione peroxidase; GRed, glutathione reductase; SOD, Superoxide dismutase; CAT, Catalase, NDM, Non-Diabetic group; DM, Diabetic group; NCAD, Non-coronary artery disease group; CAD, coronary artery disease group; SAT, subcutaneous adipose tissue; EAT, epicardial adipose tissue; [a] SAT NDM versus EAT NDM; [b] SAT DM versus EAT DM; [c] EAT NDM versus EAT DM; [d] SAT NCAD versus EAT CAD; [e] SAT CAD versus EAT CAD; [f] EAT NCAD versus EAT CAD.
